# Short-Communication: Short-Term Treatment with Taurine Prevents the Development of Cardiac Hypertrophy and Early Death in Hereditary Cardiomyopathy of the Hamster and Is Sex-Dependent

**DOI:** 10.3390/nu14163287

**Published:** 2022-08-11

**Authors:** Ghassan Bkaily, Yanick Simon, Alexandre Normand, Ashley Jazzar, Houssein Najibeddine, Abdelouahed Khalil, Danielle Jacques

**Affiliations:** 1 Department of Immunology and Cell Biology, Faculty of Medicine and Health Sciences, Université de Sherbrooke, Sherbrooke, QC J1H 5N4, Canada; 2 Department of Medicine, Faculty of Medicine and Health Sciences, Université de Sherbrooke, Sherbrooke, QC J1H 5N4, Canada

**Keywords:** taurine, cardiomyopathy, hereditary cardiomyopathy, hypertrophy, early death, heart failure, sex-dependence

## Abstract

Premature death due to heart failure is a major health problem. Taurine is a non-essential amino acid that has received much attention. However, although many studies have been carried out on the beneficial effects of taurine in cardiac pathophysiology, no studies have investigated the effect of taurine treatment on the development of hereditary cardiomyopathy (HCM) associated with hypertrophy, heart failure, and early death. This study aims to verify whether short-term treatment (20 days) with taurine in tap water prevents the development of hypertrophy and premature death in hereditary cardiomyopathy of the hamster (HCMH) of the line UM-X7.1 and if its effect is sex-dependent. Our results show that treatment for 20 days with taurine (250 mg/kg/day or 25 mg/animal/day) during the development of the hypertrophic phase (220 days old) significantly decreased (*p* < 0.01) the heart weight to body weight ratio in male HCMHs without affecting the female. During the 20 days (220–240 days old), there were nearly 40% premature deaths in non-treated males HCMHs and 50% in female HCMHs. Treatment for 20 days wholly and significantly prevented early death in both males and females HCMHs. Our results demonstrate that short-term treatment with taurine prevents the development of cardiac hypertrophy associated with HCM in a sex-dependent manner; however, it prevents early death in a sex-independent fashion. Our results suggest that taurine supplementation could be used to treat HCM.

## 1. Introduction

Premature death due to heart failure is a significant and growing health problem in North America [1]. There are nearly 5.7 million Americans aged 20 years and older living with heart failure [1,2]. Relatively similar statistics to those in the US have been reported elsewhere, and the number of cases is expected to increase over time [1,2]. Although this health problem has generally received attention, less work has been carried out on premature death associated with heart failure in inherited heart disease, such as hereditary cardiomyopathy. Recently, it has been shown that there is a deficiency of circulating taurine in dilated cardiomyopathy [3] and in patients with diabetic cardiomyopathy [4,5]. In addition, our group has shown that taurine prevents insulin-induced adult cardiomyocyte hypertrophy [6]. For a long time, taurine has been used as an anti-stress, anti-fatigue, and anti-hypertensive [7]. Many studies have shown its benefits, and as a result, taurine is now used as a supplement in infant milk and is commercially available in tablets [7,8,9]. In vivo and in vitro, taurine induces positive and negative inotropic effects on the heart, depending on the duration of supplementation [7,10,11,12]. Although its beneficial effects on the cardiovascular system are well described [1,2], those related to hypertrophy and heart failure associated with premature death remain obscure [13,14,15]. Despite the vast literature on taurine [13], no study has addressed the question of the effect of taurine and whether it is sex-dependent. It is worth noting that our study will address this aspect. In addition, there is no information available on the beneficial role of taurine in hypertrophy and heart failure associated with premature death. The primary aim of this study is to verify whether short-term treatment (20 days) with taurine in tap water prevents the development of hypertrophy and premature death in the hereditary cardiomyopathy of the hamster (HCMH), while the secondary aim is to verify whether this effect is sex-dependent.

## 2. Materials and Methods

### 2.1. Animal Model, Study Design, and Taurine

Taurine was bought from Cedarlane Labs (Burlington, ON, Canada). Chemicals whose origins are not indicated were obtained from Sigma-Aldrich (Oakville, ON, Canada).

Experiments were carried out during the beginning of the phase of hypertrophy (near 220 days old) of male and female HCMHs from the UM-X7.1 line [14,15,16,17] bred in our Faculty of Medicine animal house of the Université de Sherbrooke (Sherbrooke, QC, Canada). In the present study, all the experimental protocols for animal studies were approved by the Animal Care and Use Committee of our Faculty (the approval code is 2020–2814). As standard procedure, the HCMHs were divided, in a randomized fashion, into groups according to sex and body weight and were housed under identical conditions (24 °C and 12:12 h light: dark cycle) with free access to hamster chow and tap water in the absence or presence of taurine. The animals were cared for in accordance with the guidelines of the Canadian Council of Animal Care (Ottawa, ON, Canada). For the hypertrophy study, we divided the animals into the following groups: (1) 7 non-treated normal male hamsters; (2) 5 non-treated male HCMHs; (3) 10 taurine-treated male HCMHs; (4) 38 non-treated female normal hamsters; (5) 14 non-treated female HCMHs; and (6) 13 taurine-treated female HCMHs. For the survival experiments, the animals were divided into four groups: (1) 14 non-treated male HCMHs; (2) 16 non-treated female HCMHs; (3) 12 taurine-treated male HCMHs; and (4) 20 taurine-treated female HCMHs. The difference in the number of animals in each group was due to the availability of 220 days old male and female HCMHs in our animal breeding colony.

Two hundred twenty-day-old HCMHs were treated for 20 days with tap water containing 250 mg/kg/day (or 25 mg/animal/day) of taurine (age at the end of trials is 240 days old). The tap water was changed each morning, and the quantity consumed by the animals was measured and found to be the same in the two groups. The concentration of taurine was determined according to the supplements recommended [13]. For the study on heart weight body weight ratio, we also used a non-treated aged-matched normal hamster (Envigo, Ottawa, ON, Canada).

Mortality was assessed daily. The period of treatment for 20 days takes place at the beginning of the period of hypertrophy and early death (220 days of age) [14,16]. The animals were weighed weekly in the morning and at the end of each trial. Precautions were also taken to minimize stress on the animals used. Males and females were separated in order to avoid mating between animals. Animals of both sex that showed any signs of distress were euthanized and included in the list of premature death. In our experiments, no distress cases took place during the short-term treatment with taurine supplementation.

### 2.2. Statistical Analyses

The values are expressed as means ± standard error of the mean (S.E.M.). Mean values were compared using a one-way ANOVA test of repeated measurements for matched values, followed by the Bonferroni multiple comparison tests, where a *p*-value < 0.05 was considered significant. Statistical analyses were performed using GraphPad Prism 8.4 (GraphPad Software, La Jolla, CA, USA).

## 3. Results

### 3.1. Effect of 20 Days of Treatment with Taurine (250 mg/kg/Day or 25 mg/Animal/Day) on Heart Weight to Body Weight Ratio of Males and Females HCMHs

**Figure 1:**
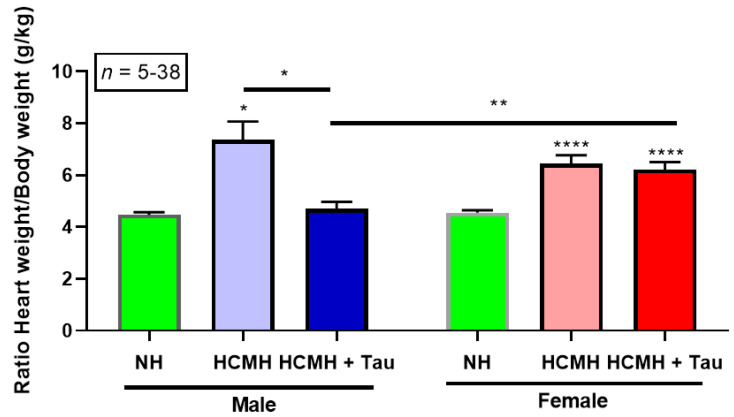
Effect of a 20-day treatment of males and females HCMHs with taurine on the heart weight/body weight ratio. Results are expressed as mean ± S.E.M. Comparisons are between untreated HCMHs and taurine-treated HCMHs. * *p* < 0.05, ** *p* < 0.001 and **** *p* < 0.0001. NH = normal hamster; HCMH = hereditary cardiomyopathic hamsters; Tau = taurine; S.E.M. = standard error of the mean; *n* = number of animals.

In this series of experiments, we tested the effect of short-term treatment (20 days) with taurine on the cardiac hypertrophy of male and female HCMHs. Figure 1 summarizes the results.

As seen in Figure 1, as expected, the heart weight/body weight ratio significantly increased in both males (normal hamster, 4.47 ± 0.10, *n* = 7 and HCMH, 7.36 ± 0.71, *n* = 5, *p* < 0.05) and females (normal hamster, 4.56 ± 0.09, *n* = 38 and HCMH, 6.44 ± 0.33, *n* = 14, *p* < 0.0001) in non-treated 240 days old hereditary cardiomyopathic hamsters. Note that the statistical significance in female HCMHs is very high compared to male HCMHs. The treatment of male HCMHs for 20 days with taurine completely prevented the development of hypertrophy in HCMH (4.70 ± 0.27, *n* = 10) to a level similar to non-treated aged-matched normal male hamsters (4.47 ± 0.10, *n* = 7). 

Twenty days of treatment with taurine in 220 days old female HCMH did not affect the heart weight/body weight ratio (HCMH, 6.44 ± 0.33, *n* = 14 and HCMH + taurine, 6.21 ± 0.29, *n* = 13). However, as can also be seen in this figure, there are significant differences (*p* < 0.001) between taurine-treated female (6.21 ± 0.29, *n* = 13) and male (4.70 ± 0.27, *n* = 10) HCMHs. Figure 1 clearly shows that the cardiac hypertrophy of female cardiomyopathic hamsters was completely unaffected by 20 days of treatment with taurine.

### 3.2. Effect of 20 Days of Treatment with Taurine (250 mg/kg/Day or 25 mg/Animal/Day) on the Early Death of 220-Day-Old Male and Female HCMHs

**Figure 2:**
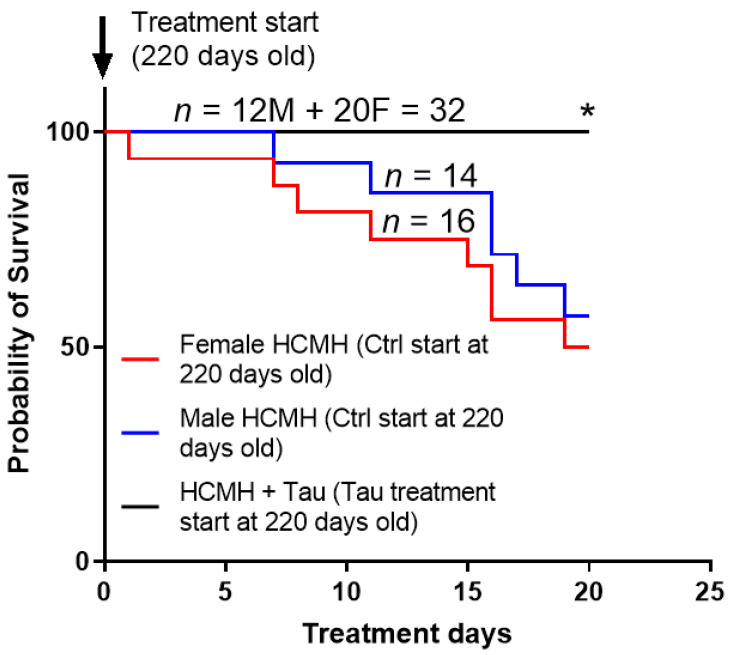
Curves showing the effects of preventive 20 days treatment (black plot) with taurine on the survival of male (M) and female (F) 220-day-old hereditary cardiomyopathic hamsters (HCMHs). The arrows indicate the age of HCMHs at the beginning and the end of the preventive treatment. Ctr = control non-treated animals; Tau = taurine-treated animals. Values are expressed as percent survival, and *n* is the number of animals. * *p* < 0.05.

In this series of experiments, we verified whether 20 days of treatment with taurine (250 mg/kg/day or 25 mg/animal/day) during the hypertrophic phase of hereditary cardiomyopathy [14,17] affected early death of 220-day-old HCMHs. In addition, we wanted to verify whether the effect of short-term treatment with taurine on survival was sex-dependent. Figure 2 summarizes the results. As can be seen in this figure, there is a 42.9% mortality in non-treated male HCMHs (however, there was higher mortality (50%) in non-treated female HCMHs during the development of hypertrophy (220–240 days old). Furthermore, the progression of early death is higher in non-treated female HCMHs than in non-treated aged-matched male HCMHs. As can also be seen in this figure, treatment for 20 days with taurine significantly and completely prevented early death in both male and female HCMHs (*p* < 0.05).

## 4. Discussion

Nowadays, taurine is present in almost all energy drinks. The beneficial effects of taurine have been demonstrated in many diseases [18,19,20,21] with the exception of hereditary cardiomyopathy. Among other things, it promotes the lowering of serum low-density lipoprotein (LDL), reduces atherosclerosis progression, and protects against myocardial ischemia-reperfusion injury [7,8,9,22,23].

Although it is recognized that the tissue content of taurine is sex-dependent [18,19], all studies on this amino acid have been carried out mainly, if not exclusively, on the male, without considering the female under normal or pathological conditions, particularly at the cardiac level.

Our results showed for the first time that taurine supplements over a short period of 20 days completely prevented the development of hypertrophy associated with early death in hereditary cardiomyopathic males without affecting hypertrophy in females. This effect shows that short-term treatment with taurine is sex-dependent. The absence of the effect of short-term treatment with taurine on the hypertrophy of female cardiomyopathic hamsters could be due in part to the higher severity of the disease in females compared to males. The severity of the disease in females compared to males can be seen in Figure 2. Our results also showed that the mortality seems to be more frequent and elevated in females than males HCMHs. This aspect should be clarified in the future using long-term treatment with taurine as was performed previously using the sodium/hydrogen exchanger blocker [16,17].

Contrary to hypertrophy, survival during the 20 days of treatment with taurine was found to be sex-independent. This difference in the effect of short-term treatment with taurine in preventing early death but not the hypertrophy in females HCMH could also be due to the difference between the mechanism responsible for the development of hypertrophy and that responsible for premature death in females but not necessarily in males. This aspect should be verified in the future. Finally, we recently showed that endocardial endothelium cells that cover the ventricle cavities [24,25] undergo hypertrophy during the development of HCM [24]. Whether the short-term treatment of taurine prevents the development of endocardial endothelium hypertrophy is to be explored.

## 5. Conclusions

In conclusion, this is the first study to show that supplementation with taurine in drinking water alone can, even in the short term, prevent early death in hereditary cardiomyopathy in both males and females. However, while the prevention of cardiac hypertrophy associated with hereditary cardiomyopathy through short-term treatment with taurine seems to be sex-dependent, this may not be the case with long-term taurine treatment. This aspect awaits discovery. Nevertheless, our results provide hope for the treatment of hereditary cardiomyopathy leading to early death, such as in Duchenne and Becker muscular dystrophies [14,15,16,17,26]. More studies are needed to explore this new treatment for such a heart-killing disease and to understand the mechanisms by which taurine prevents the development of hereditary cardiomyopathy associated with early death.

## 6. Limitations of the Study

We have to mention that our pilot study is limited to the sample of hamsters studied. There is no doubt that our pilot study may have some other limitations and, more particularly, whether life-long supplementation with taurine will prevent all the phases of hereditary cardiomyopathy, including early death, and whether is it is sex-dependent.

## Data Availability

The data presented in this study are available on request from the corresponding author.

## References

[1] Blair J.E., Huffman M., Shah S.J. (2013). Heart Failure in North America. Curr. Cardiol. Rev..

[2] Roger V.L., Go A.S., Lloyd-Jones D.M., Benjamin E.J., Berry J.D., Bordenm W.B., Bravata D.M., Dai S., Ford E.S., Fox C.S. (2012). American Heart Association Statistics Committee and Stroke Statistics Subcommittee. 2012. Heart disease and stroke statistics--2012 update: A report from the American Heart Association. Circulation.

[3] Ontiveros E.S., Whelchel B.D., Yu J., Kaplan J.L., Sharpe A.N., Fousse S.L., Crofton A.E., Fascetti A.J., Stern J.A. (2020). Development of plasma and whole blood taurine reference ranges and identification of dietary features associated with taurine deficiency and dilated cardiomyopathy in golden retrievers: A prospective, observational study. PLoS ONE.

[4] Franconi F., Bennardini F., Mattana A., Miceli M., Ciuti M., Mian M., Gironi A., Anichini R., Seghieri G. (1995). Plasma and platelet taurine are reduced in subjects with insulin-dependent diabetes mellitus: Effects of taurine supplementation. Am. J. Clin. Nutr..

[5] Franconi F., Di Leo M.A., Bennardini F., Ghirlanda G. (2004). Is taurine beneficial in reducing risk factors for diabetes mellitus?. Neurochem. Res..

[6] Jazzar A., Jacques D., Bkaily G. (2021). Insulin-Induced Cardiomyocytes Hypertrophy That Is Prevented by Taurine via β-alanine-Sensitive Na^+^-Taurine Symporter. Nutrients.

[7] Schaffer S., Kim H.W. (2018). Effects and Mechanisms of Taurine as a Therapeutic Agent. Biomol. Ther..

[8] Xu Y.-J., Arneja A.S., Tappia P.S., Dhalla N.S. (2008). The potential health benefits of taurine in cardiovascular disease. Exp. Clin. Cardiol..

[9] Abebe W., Mozaffari M.S. (2011). Role of taurine in the vasculature: An overview of experimental and human studies. Am. J. Cardiovasc. Dis..

[10] Bkaily G., Haddad G., Jaalouk D., Gros-Louis N., Benchekroun M.T., Naik R., Pothier P., D’Orléans-Juste P., Bui M., Wang S. (1996). Modulation of Ca^2+^ and Na^+^ transport by taurine in heart and vascular smooth muscle. Adv. Exp. Med. Biol..

[11] Bkaily G., Jaalouk D., Haddad G., Gros-Louis N., Simaan M., Naik R., Pothier P. (1997). Modulation of cytosolic and nuclear Ca^2+^ and Na^+^ transport by taurine in heart cells. Mol. Cell. Biochem..

[12] Bkaily G., Jaalouk D., Sader S., Shbaklo H., Pothier P., Jacques D., D’Orléans-Juste P., Cragoe E.J., Bose R. (1998). Taurine indirectly increases [Ca]i by inducing Ca^2+^ influx through the Na^+^-Ca^2+^ exchanger. Mol. Cell. Biochem..

[13] Bkaily G., Jazzar A., Normand A., Simon Y., Al-Khoury J., Jacques D. (2020). Taurine and cardiac disease: State of the art and perspectives. Can. J. Physiol. Pharmacol..

[14] Bkaily G., Jacques D. (2017). Na^+^-H^+^ exchanger and proton channel in heart failure associated with Becker and Duchenne muscular dystrophies. Can. J. Physiol. Pharmacol..

[15] Jasmin G., Proschek L. (1982). Hereditary polymyopathy and cardiomyopathy in the Syrian hamster. I. Progression of heart and skeletal muscle lesions in the UM-X7.1 line. Muscle Nerve.

[16] Bkaily G., Chahine M., Al-Khoury J., Avedanian L., Beier N., Scholz W., Jacques D. (2015). Na^+^-H^+^ exchanger inhibitor prevents early death in hereditary cardiomyopathy. Can. J. Physiol. Pharmacol..

[17] Chahine M., Bkaily G., Nader M., Alkhoury J., Jacques D., Beier N., Scholz W. (2005). NHE-1-dependent intracellular sodium overload in hypertrophic hereditary cardiomyopathy: Prevention by NHE-1 inhibitor. J. Mol. Cell. Cardiol..

[18] Jacques D., Tong Y., Shen S.H., Quirion R. (1998). Discrete distribution of the neuropeptide Y Y5 receptor gene in the human brain: An in situ hybridization study. Mol. Brain Res..

[19] Yamori Y., Liu L., Ikeda K., Miura A., Mizushima S., Miki T., Nara Y., Who-Cardiovascular Disease and Alimentary Comprarison (CARDIAC) Study Group (2001). Distribution of twenty-four hour urinary taurine excretion and association with ischemic heart disease mortality in 24 populations of 16 countries: Results from the WHO-CARDIAC study. Hypertens Res..

[20] Shao A., Hathcock J.N. (2008). Risk assessment for the amino acids taurine, l-glutamine and l-arginine. Regul. Toxicol. Pharmacol..

[21] Chesney R.W., Han X., Patters A.B. (2010). Taurine and the renal system. J. Biomed. Sci..

[22] Lambert I.H., Kristensen D.M., Holm J.B., Mortensen O.H. (2014). Physiological role of taurine—From organism to organelle. Acta Physiol..

[23] De Luca A., Pierno S., Camerino D.C. (2015). Taurine: The appeal of a safe amino acid for skeletal muscle disorders. J. Transl. Med..

[24] Jacques D., Bkaily G. (2019). Endocardial endothelial cell hypertrophy takes place during the development of hereditary cardiomyopathy. Mol. Cell. Biochem..

[25] Jacques D., Malak N.A.A., Sader S., Perreault C. (2003). Angiotensin II and its receptors in human endocardial endothelial cells: Role in modulating intracellular calcium. Can. J. Physiol. Pharmacol..

[26] Al-Khoury J., Jacques D., Bkaily G. (2022). Hypotension in hereditary cardiomyopathy. Pflug. Arch..

